# Oral mucosal lesions in a Chilean elderly population: A retrospective study with a systematic review from thirteen countries

**DOI:** 10.4317/jced.53427

**Published:** 2017-02-01

**Authors:** César Rivera, Daniel Droguett, María-Jesús Arenas-Márquez

**Affiliations:** 1 Department of Basic Biomedical Sciences, Faculty of Health Sciences, University of Talca (UTALCA), Talca, Chile; 2 Department of Oral Diagnosis, School of Dentistry (FOP), University of Campinas (UNICAMP), Piracicaba, São Paulo, Brazil; 3 Department of Stomatology, Faculty of Health Sciences, University of Talca (UTALCA), Talca, Chile; 4 Gerontology Program, Faculty of Medical Sciences, University of Campinas (UNICAMP), Campinas, São Paulo, Brazil

## Abstract

**Background:**

The oral examination is an essential part of the multidisciplinary medical care in elderly people. Oral mucosal lesions and normal variations of oral anatomy (OMLs) are very common in this people, but few studies have examined the frequency and prevalence of these conditions worldwide and less in Chile. The aim of this research was to evaluate the frequency of OMLs in a Chilean elderly population.

**Material and Methods:**

It was conducted a retrospective study (Talca, Chile). Two hundred seventy-seven OMLs were classified in groups and anatomical sites. In order to contextualize our numbers, we made a systematic review using Publish or Perish software, Google Scholar and InteractiVenn.

**Results:**

The most prevalent OMLs groups were soft tissue tumors, epithelial pathology, facial pain and neuromuscular diseases, and dermatologic diseases. The most frequent OMLs included irritation fibroma (30 patients, 10.8%), hemangioma (20, 7.2%), burning mouth syndrome (20 cases, 7.2%), oral lichen planus (12, 4.3%) and epulis fissuratum (12, 4.3%). In the systematic review, 75 OMLs were relevant and the more studied pathologies were traumatic ulcerations (11 of 15 articles), oral lichen planus (10/15), irritation fibroma, melanotic pigmentations, and recurrent aphthous stomatitis (9/10, respectively). Considering all included articles, most frequent OMLs in elderly people included denture-related stomatitis (13.3%), irritation fibroma (8.7%) and fissured tongue (6.3%).

**Conclusions:**

The results reflect the frequency of OMLs diagnosed in a specialized service in south of Chile and many countries around the world. These numbers will allow the establishment of preventive politics and adequacy of the clinical services.

** Key words:**Oral mucosal lesions, elderly people, Chilean population, frequency, systematic review.

## Introduction

Aging involves multiple aspects, because of this a multidisciplinary team -incorporating relevant clinical gerodontology- should provide oral health care (1). Older people perceive oral health as being important to life quality in a variety of ways (2). Disorders affecting the mouth soft tissues, salivary glands and mucosa are common oral health problems that can affect the quality of life (3).

To maintain the oral health, oral examination by a dental surgeon is an essential part of the multidisciplinary medical care in this group of individuals (4). Oral mucosal lesions and normal variations of oral anatomy (OMLs) are very common in this age group, but few studies have examined the frequency and prevalence of these conditions worldwide and less in Chile (5,6). According to the Chilean Institute of Statistics (INE), our country has a very advanced process of aging population and by 2020 it is expected that 17.3% of Chileans will be in this age group (7).

Hence, the aim of this research was to determine the frequency OMLs from a Chilean population over a 14- year period. Additionally, we made a systematic review to contextualize our results and summarize the conclusions of published studies around the world.

## Material and Methods

-Patients. The School of Dentistry of University of Talca is the only dental school in the Maule region and offers dental services to the public of in south-central of Chile. We conducted a retrospective study with a convenience sampling of 277 elderly patients from the oral pathology and medicine service (a small clinic, which operates twice a week). Our study covered a period of 14 years, from March 2001 to December 2014 (clinicopathological characteristics are presented in  Table 1,  Table 1 continue). The minimal inclusion criteria for cases was a presence of one clear clinical and histopathological diagnosis. This research has been conducted according to the principles expressed in the Declaration of Helsinki. (version 2002). We obtained the informed consent of each individual; in order to preserve confidentiality, database was encrypted. University of Talca Review Board approved all procedures (2014-027).

**Table 1:**
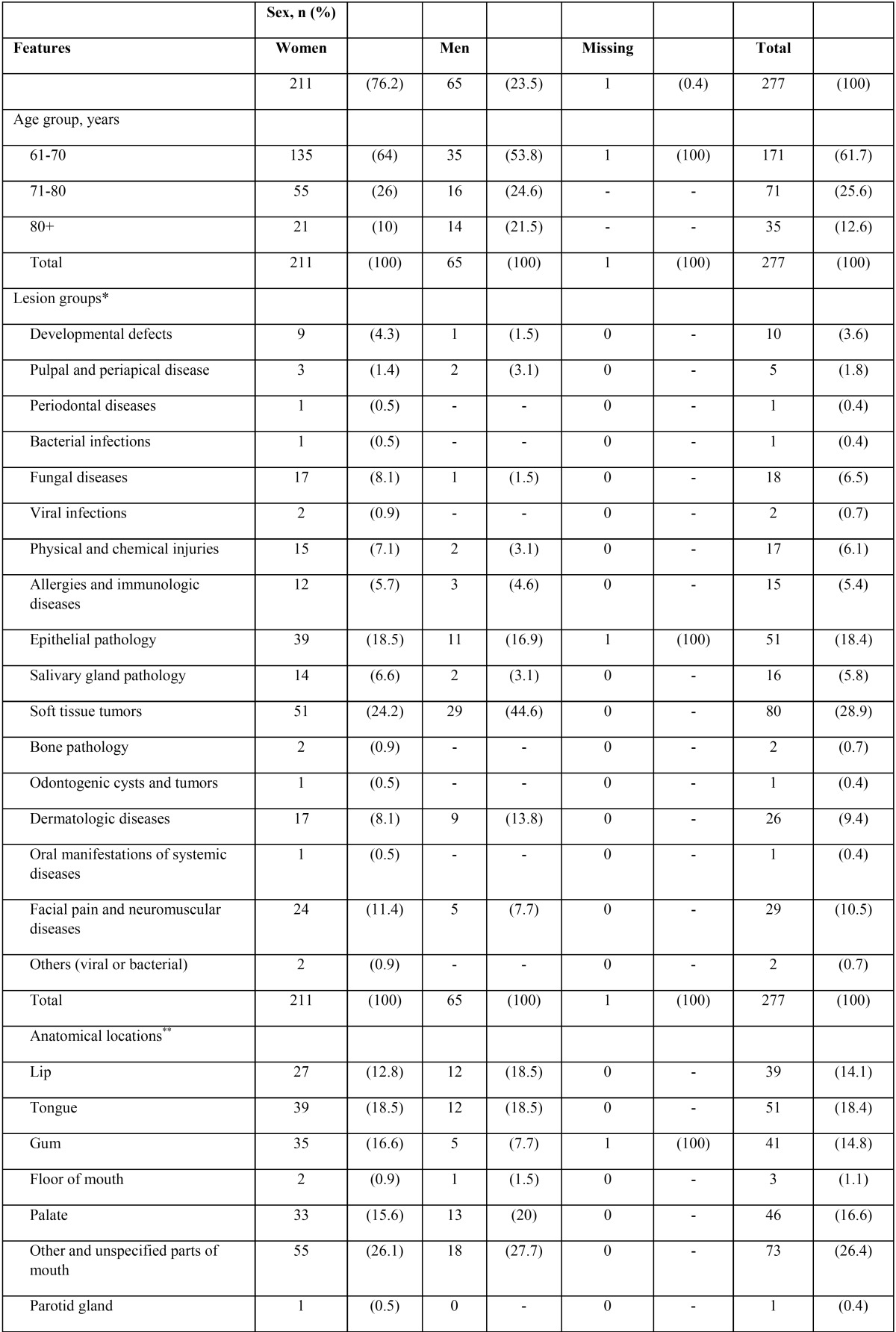
Clinical and pathological characteristics of Chilean elderly patients with oral mucosal lesions and normal variations of oral anatomy (OMLs, n=277).

**Table 1 continue:**
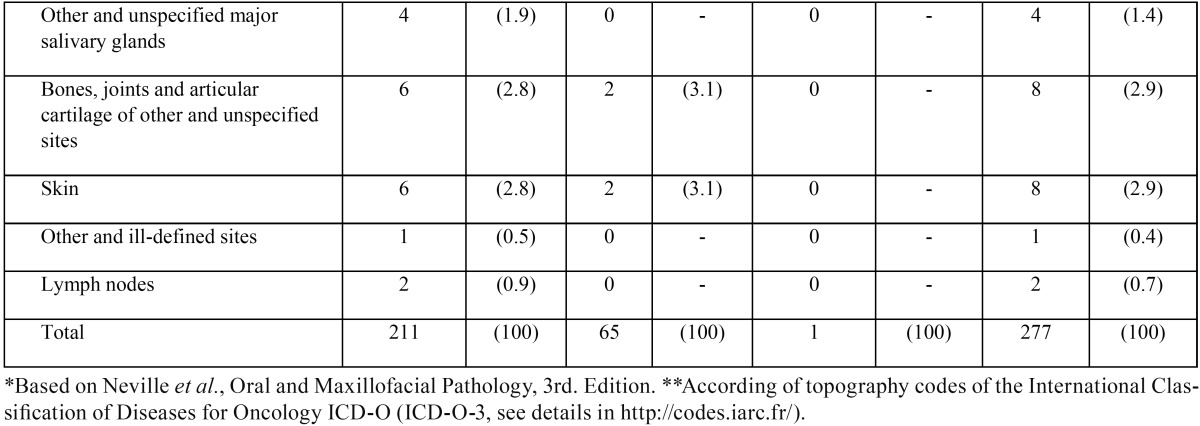
Clinical and pathological characteristics of Chilean elderly patients with oral mucosal lesions and normal variations of oral anatomy (OMLs, n=277).

-Outcomes. The main outcomes evaluated relating to the OMLs were the type of alteration and site of occurrence. OMLs were classified by type according to Neville *et al.* textbook (8). Anatomical sites were reported, according to the International Classification of Diseases for Oncology (ICD-O-3, see details in http://codes.iarc.fr). The results were informed following the STROBE statement.

-Systematic review. To contextualize our results and with the aim of answering the question, “What are the OMLs frequency/prevalence in elderly people?” a systematic literature search based on relevant papers (ranking) was performed to provide the best results. To identify all primary research studies that evaluated OMLs in oral cavity of elderly people, we searched the Google Sholar (GS) data source up to March 26, 2016, using Publish or Perish (PoP) software (http://www.harzing.com/resources.htm). The search strategy were conducted using guidelines published previously in the software webpage and terms: “oral mucosal lesions” (all the words) and “elderly people adult aged” (any of the words), using “title words only filter” and English as language (supplementary figure 1, DOI: 10.6084/m9.figshare.3573681.v1). Duplicate articles were eliminated with CleanPoP (http://cleanpop.ifris.org).

-Inclusion criteria and data extraction. Articles were included if they examined OMLs prevalence/frequency in elderly patients. Manuscripts were included irrespective of studies’ design. We also did not have any limitation on the sample size of the study. Two readers (CR and MJA-M) selected articles for inclusion. Investigators reviewed all eligible studies and carefully extracted study characteristics, including article citation information, PubMed ID, country, study design, sample size and number of lesions events. The results were informed following the PRISMA statement.

-Logical relations and statistical analysis. To explore possible logical relations between data sets, we plotted logic diagrams using InteractiVenn (http://www.interativenn.net). The data were analyzed with descriptive statistics (frequency and percent) using Microsoft Excel 2013 (Microsoft Corporation, Seattle, USA) and SPSS statistical package 17 for Windows (IBM, Chicago, USA).

## Results

-Characteristics of study participants. Complete register can be accessed at supplementary file (S1, 10.6084/m9.figshare.3573681.v1). Females represent 72.6% of the sample. The mean age ± standard deviation of the patients was 70.4±0.5 years (ranging from 61 to 97 years old) and most patients were in their sixties to seventies. Seventy-two OMLs were found and the most prevalent groups were soft tissue tumors (80, 28.9%), epithelial pathology (51, 18.4%), facial pain and neuromuscular diseases (29, 10.5%), and dermatology diseases (26, 9.4%).  Table 1 shows the distribution of patients, according to gender, age, lesion groups and anatomical sites.

-Top five OMLs in Chilean elderly patients. Based on frequency, lesions were listed as “top five” ( Table 2, OMLs with a frequency between 9 and 30). See details on the supplementary archive S1 (DOI: 10.6084/m9.figshare.3573681.v1). The most frequent OMLs included irritation fibroma (30 patients, 10.8%), hemangioma (20, 7.2%), burning mouth syndrome (BMS, also termed glossodynia or glossopyrosis, 20 cases, 7.2%), oral lichen planus (OLP, 12, 4.3%) and epulis fissuratum (12, 4.3%). Oral cavity squamous cell carcinoma (OSCC) represented 1.8% of the sample (5 cases). In women, OMLs were mostly represented by irritation fibroma (19, 9%), BMS (18, 8.5%) and hemangioma (12, 5.7%). Men’s ranking was lead by irritation fibroma (11, 16.9%), hemangioma (8, 12.3%), OLP (4, 6.2%) and vascular malformation (4, 6.2%).

**Table 2:**
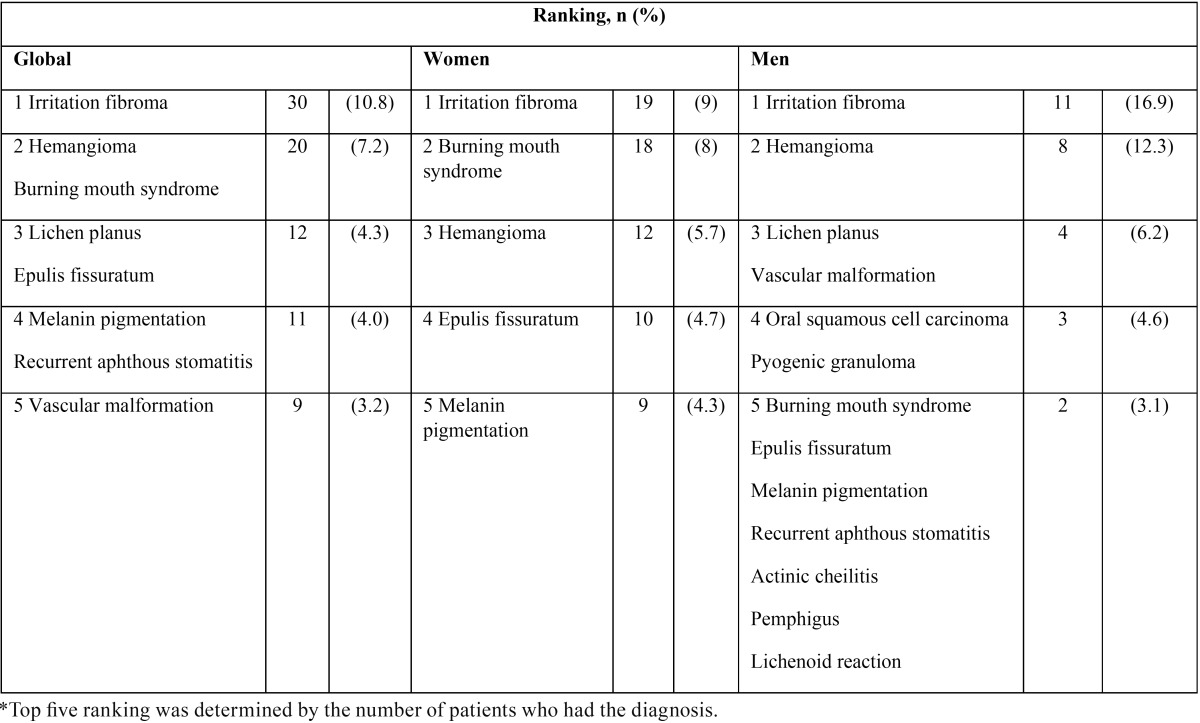
Top five OMLs* in Chilean elderly patients.

-OMLs according anatomical sites. In general, OMLs affected more frequently “unspecified parts of mouth” (ICD-O-3 C02 code, including cheek, vestibule and retromolar area, 73 cases, 26.4%), tongue (51, 18.4%) and palate (46, 16.6%). Irritation fibroma commonly affects the cheek mucosa (10, 33.3%). Burning mouth syndrome (BMS) was mainly found on the tongue-NOS (6, 30%) and mouth-NOS (12, 60%). Oral lichen planus (OLP) was principally diagnosed on the cheek mucosa (6, 50%). (Supplementary Table 1, DOI: 10.6084/m9.figshare.3573681.v1)

**Figure 1:**
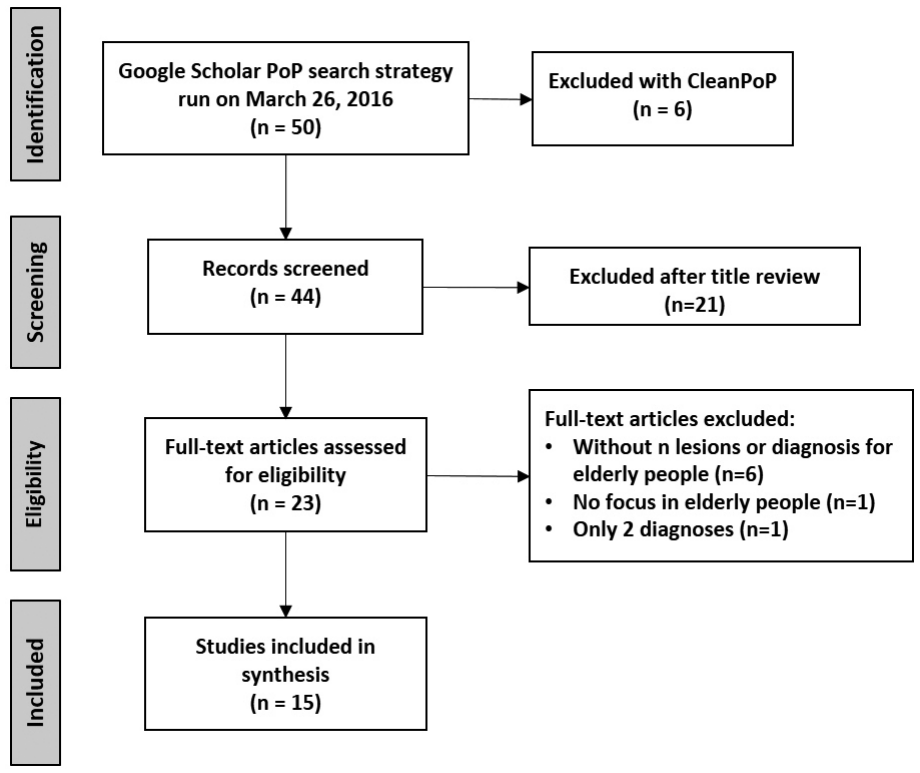
PRISMA fluxogram for the selection of articles. We searched the Google Scholar data source using PoP software, based on terms: “oral mucosal lesions” (all the words) and “elderly people adult aged” (any of the words), using title words only filter and English language. Duplicate articles were eliminated using CleanPoP.

-Systematic review: description of the literature. Figure 1 displays the PRISMA fluxogram of the search strategy. The strategy identified 50 suitable results, from which 27 were excluded from title/abstract review during the screen, as they did not meet the eligibility criteria. Full text articles were obtained for 23 studies. Eight were excluded for differing reasons. Finally, we included 15 articles (5,6,9-21) (See details at S2, DOI: 10.6084/m9.figshare.3573681.v1). The selected studies were screened, and specific study characteristics were recorded (including top five frequency/prevalence diagnoses). Supplementary  Table 2 (DOI: 10.6084/m9.figshare.3573681.v1) displays the characteristics of the studies that satisfied the criteria of eligibility. All summarized items were indexed in MEDLINE/PubMed. Included studies were conducted in Chile, Finland, Denmark, China, USA, Turkey, Israel, Malaysia, Brazil, Iran, Croatia, Nigeria and Yemen. Variable cohort sizes were reported, ranging from 126 to 1,515 patients. N, age ranges, lesions prevalence percent, and top five diagnoses were extracted. There were methodological discrepancies between the studies, so it was not possible to analyze the results using meta-analysis.

**Figure 2:**
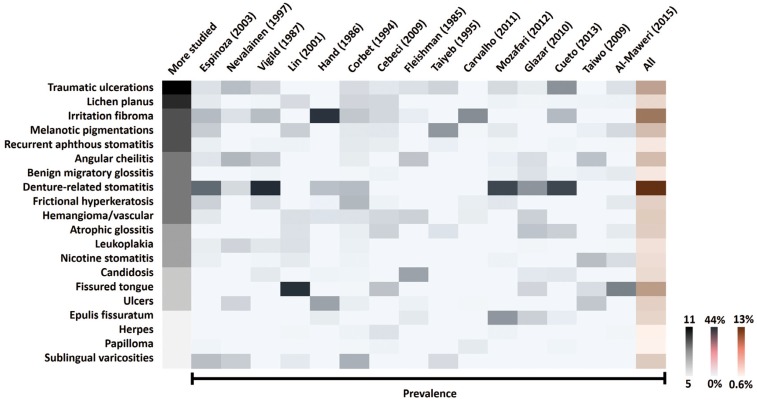
Systematic review: heat map for more studied lesions. OMLs reported in ≥5 articles. The heat map combines the most frequent OMLs (traumatic ulcerations to sublingual varicosities), rates by number of articles (gray bar) and prevalence across studies, ordered by Google Scholar rank (Espinoza to Al-Maweri). Higher numbers represent intense and saturated colors (extreme values on the right-hand side) (see S2 for details). More studied lesions were traumatic ulcerations and oral lichen planus. The most prevalent lesions were denture-related stomatitis and irritation fibroma.

-Systematic review: top five OMLS around the world. Based on frequency or prevalence (percent), we listed the “top five” OMLs (Supplementary Table 3, DOI: 10.6084/m9.figshare.3573681.v1) in all included studies. The most frequent diseases are reactive OMLs. To better understand how diagnoses behave across studies, we made a heat map (Fig. 2). As a synthesis, the more studied were traumatic ulcerations (11 of 15 articles), OLP (10/15), irritation fibroma, melanotic pigmentations, and recurrent aphthous stomatitis (9/10, respectively). Considering all articles (frequencies were added), the most frequent OMLs in elderly people included denture-related stomatitis (13.3%), irritation fibroma (8.7%) and fissured tongue (6.3%).

**Figure 3:**
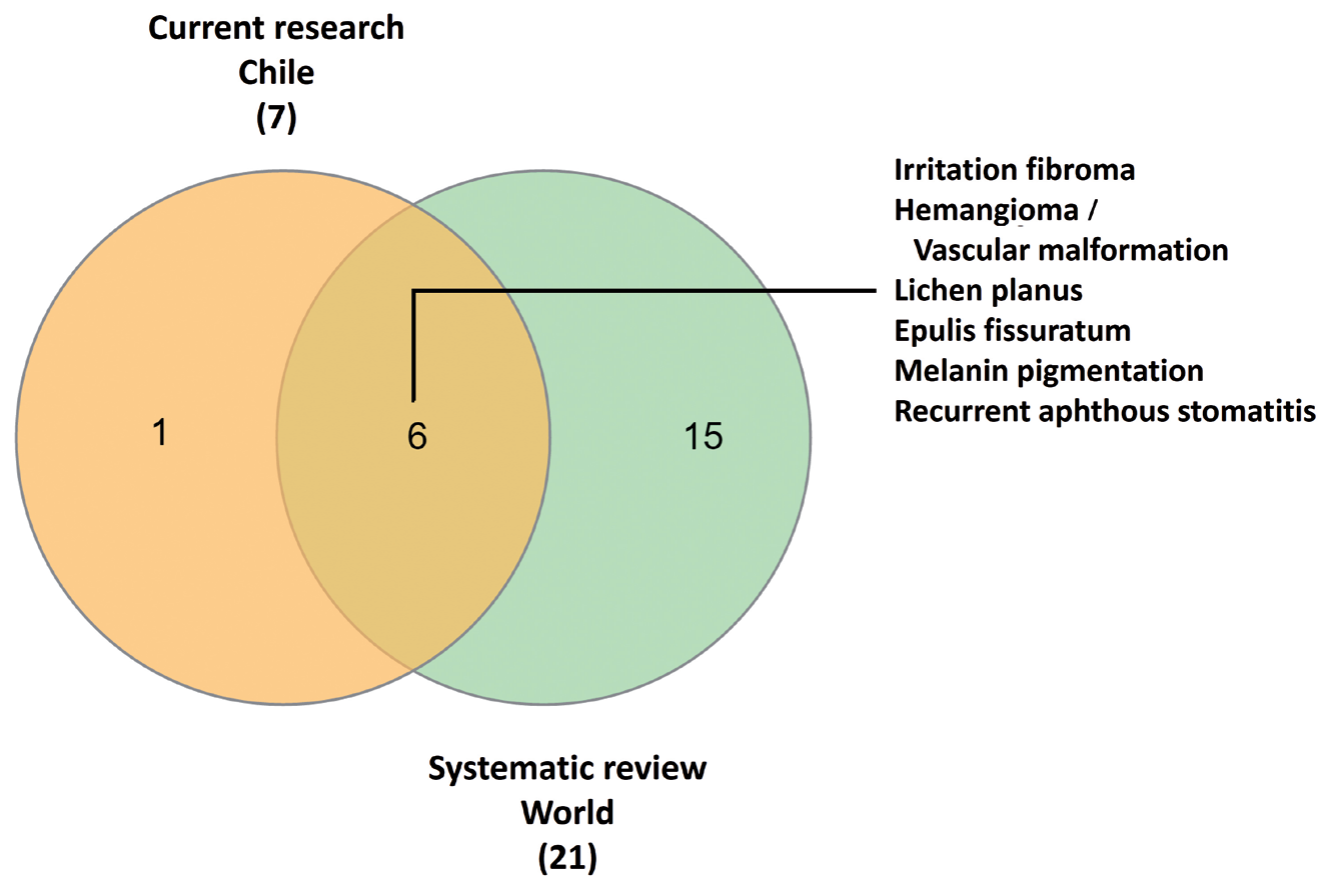
Venn diagram for the most studied and frequent OMLs. Comparison of ranked lists for current study (Chilean elderly patients) and systematic review (studies around the world). Seven of 8 diagnoses (87.5%) in our top five raking were shared with selected studies. This Chilean sample is highly compatible with global data.

-Chilean patients and the systematic review. To understand the similarity between our sample and the systematic review, we plotted a Venn diagram (Fig. 3). Seven (7/8, 87.5%) of our top five OMLs are consistent with the most studied OMLs in elderly patients around the world: irritation fibroma, hemangioma/vascular malformation, OLP, epulis fissuratum, melanin pigmentation and recurrent aphthous stomatitis (RAS).

## Discussion

This research was a convenience sampling retrospective study in the elderly patients in Chile to determine the frequency of OMLs. To the best of our knowledge, this is the third study to provide data of OMLs among an elderly population in Chile. Comparing our results with previous articles from our country (all cross-sectional) (5,6), we shared one of the 5 most relevant oral pathologies: irritation fibroma. With the exception of BMS, Espinoza *et al.* and Cueto *et al.* report all our top five OMLs previously.

In this study, elderly women have most OMLs. This may represent a source of potential bias or imprecision, because in women are more considerate of their oral health and are more concerned to prevent oral problems than men (22).

In our sample, the most frequent OMLs included irritation fibroma, hemangioma, BMS, OLP and epulis fissuratum. Since this report is not a prevalence cross-sectional study, we made a systematic review of relevant articles using GS, PoP software, and InteractiVenn to contextualize our results.

Irritation fibroma (traumatic fibroma or fibrous hyperplasia) were the most common type of OML identified in this study, the third most studied disease in the literature review and the second most frequent OML in elderly around the world (it was only surpassed by denture-related stomatitis). Is a soft tissue mass usually found on buccal mucosa along the line of occlusion (accidental biting), which is consistent with our observations. The color is usually the same as the surrounding mucosa and the consistency is surprisingly soft. Patients are generally aware of the lesion being present months to years with little change. Histologically, they exhibit fibrous hyperplasia that is collagenous and acellular (23). It is also interesting to mention that irritation fibroma is a global problem, but with an easily clinical management.

The second most common diagnosis in this research was oral hemangioma. This neoplasia was the fourth most studied disease in the literature review. Hemangioma is a benign proliferation of endothelial cells. It presents as a red macula, papule or nodule, depending on the congestion degree and on how deep it is in the tissue (24). The tumor may be slowly progressive. Oral lesions generally appear on the lips, buccal mucosa and tongue (25).

In our top five ranking (in the same position of hemangioma), BMS was the second most common diagnosis in Chilean elderly patients. This syndrome is characterized by a burning sensation in the tongue or other sites of the oral cavity, usually in the absence of clinical and laboratory findings (26). This condition was not among the most diagnosed in the systematic review, which opens interesting questions in our sample.

OLP was the third most common diagnosis in our patients. This pathology was the second most studied disease in the literature review. It shows up as a big challenge for elderly populations. OLP is a chronic inflammatory, T-cell-mediated autoimmune oral mucosal disease (potentially malignant disorder) with unclear etiology. The clinical management of OLP poses considerable difficulties to dental surgeons (27). The red, inflamed lesions and open sores of oral lichen planus can cause a burning sensation or pain. The white, lacy patches may not cause discomfort when they appear on the cheek mucosa but may be painful when they involve the tongue (28). In our population, OLP affects mostly cheek mucosa.

Epulis fissuratum (inflammatory fibrous hyperplasia or denture-induced fibrous hyperplasia) closes the top three positions of our Chilean ranking, also is among the most prevalent diseases reported by the systematic review. This lesion attributed to reactive tissue response to chronic irritation and trauma caused by a badly fitted prosthesis (29).

The major limitations of our research are related to firstly, the patients treated by the oral pathology and medicine service at The School of Dentistry of University of Talca may not be able to represent “an urban elderly population of Chile”. Secondly, the sample size is also too small compared to the urban population of Chile, the frequency of OMLs depends entirely on the clinical queries received. We conducted a systematic review using GS. The major disadvantage of GS resides in the fact that there is no distinction between a paper in a well-known journal, a book, a scientific report, etc. However, concerning the numbers of articles, number of citations, or the h-index, the contribution of the GS database is clearly visible (30). We were hoping that denture-related stomatitis were the most common oral lesion; however, in our university, lesions associated with dentures are treated in the oral rehabilitation clinic.

The importance of the oral medicine specialist is therefore crucial to the management of patients presenting OMLs. As life expectancy in Chile is around 80 years and continues to rise (1), is necessary have regional centers with oral pathologists to diagnose and manage these diseases. Our findings contribute substantially to the body of OMLs literature in elderly patients, which might provide valuable in planning future studies and interventions in these people. Future prevalence studies in this and other regions are needed. We believe that conducting a systematic review, as an integral part of this research, was essential to avoid only number comparisons in the discussion section. As a conclusion, relevant OMLs (including Chilean dataset and systematic review) in elderly people were irritation fibroma, hemangioma, BMS, OLP, epulis fissuratum, traumatic ulcerations, denture-related stomatitis, melanotic pigmentations, recurrent aphthous stomatitis and fissured tongue. The results reflect the frequency and prevalence of OMLs diagnosed in elderly patients and will allow the establishment of preventive politics, adequacy of the clinical services and dentistry curricular emphasis.
